# Translocase of the Outer Mitochondrial Membrane 40 Is Required for Mitochondrial Biogenesis and Embryo Development in *Arabidopsis*

**DOI:** 10.3389/fpls.2019.00389

**Published:** 2019-04-02

**Authors:** Ying Hu, Wenxuan Zou, Zhiqin Wang, Yuqin Zhang, Yuanyuan Hu, Jie Qian, Xuan Wu, Yafang Ren, Jie Zhao

**Affiliations:** State Key Laboratory of Hybrid Rice, College of Life Sciences, Wuhan University, Wuhan, China

**Keywords:** *Arabidopsis*, mitochondria biogenesis, translocase of the outer mitochondrial membrane 40 (TOM40), embryo development, pattern formation

## Abstract

In eukaryotes, mitochondrion is an essential organelle which is surrounded by a double membrane system, including the outer membrane, intermembrane space and the inner membrane. The translocase of the outer mitochondrial membrane (TOM) complex has attracted enormous interest for its role in importing the preprotein from the cytoplasm into the mitochondrion. However, little is understood about the potential biological function of the TOM complex in *Arabidopsis*. The aim of the present study was to investigate how *AtTOM40*, a gene encoding the core subunit of the TOM complex, works in *Arabidopsis.* As a result, we found that lack of *AtTOM40* disturbed embryo development and its pattern formation after the globular embryo stage, and finally caused albino ovules and seed abortion at the ratio of a quarter in the homozygous *tom40* plants. Further investigation demonstrated that *AtTOM40* is wildly expressed in different tissues, especially in cotyledons primordium during *Arabidopsis* embryogenesis. Moreover, we confirmed that the encoded protein AtTOM40 is localized in mitochondrion, and the observation of the ultrastructure revealed that mitochondrion biogenesis was impaired in *tom40-1* embryo cells. Quantitative real-time PCR was utilized to determine the expression of genes encoding outer mitochondrial membrane proteins in the homozygous *tom40-1* mutant embryos, including the genes known to be involved in import, assembly and transport of mitochondrial proteins, and the results demonstrated that most of the gene expressions were abnormal. Similarly, the expression of genes relevant to embryo development and pattern formation, such as SAM (shoot apical meristem), cotyledon, vascular primordium and hypophysis, was also affected in homozygous *tom40-1* mutant embryos. Taken together, we draw the conclusion that the *AtTOM40* gene is essential for the normal structure of the mitochondrion, and participates in early embryo development and pattern formation through maintaining the biogenesis of mitochondria. The findings of this study may provide new insight into the biological function of the TOM40 subunit in higher plants.

## Introduction

In the past decades, it has been widely studied that the process of embryogenesis in *Arabidopsis* is highly programmed and controlled by an intricate network of gene expression (ten Hove et al., 2015). The single-celled zygote (fertilized egg cell) initiates cell division and differentiation to produce a mature embryo, while the triploid primary endosperm nucleus (fertilized central polar nucleus) develops into a syncytial endosperm and subsequently undergoes cellularization to form endosperm (Hamamura et al., 2012; Sprunck and Dresselhaus, 2015). The endosperm nourishes the embryo and determines the size of ovule (Lafon-Placette and Köhler, 2014). The onset of early embryogenesis forms the precursors for all stem cells and major tissues (Capron et al., 2009; Lau et al., 2012; Bleckmann et al., 2014). Previous research focused on the molecular mechanisms in the formation of the tissue primordium, and demonstrated that a large number of genes were required for the process of embryo development and morphogenesis in *Arabidopsis* (Tzafrir et al., 2004; Meinke et al., 2008; Palovaara et al., 2017), which helped to explore the relationship between some critical biological events and embryo development.

The mitochondrion is an essential organelle derived from an endosymbiotic event two billion years ago, and is the main source of ATP to sustain cellular viability and activity (Gray et al., 1999; Cavalier-Smith, 2009; Alcock et al., 2010). In eukaryotes, a small portion of mitochondrial proteins are encoded by the mitochondrial genes, while most proteins are encoded by the nuclear genome. The proteins are synthesized in the cytoplasm and then imported into the mitochondria (Adams and Palmer, 2003; Crimi and Rigolio, 2008; Lithgow and Schneider, 2010). The protein transport process is mediated by the mitochondrial double-membrane system, including the outer membrane (OM), intermembrane space (IMS) and the inner membrane (IM). The translocase of the outer mitochondrial membrane (TOM) complex functions as the entry gate on the OM, it can recognize the preproteins synthesized in the cytosol and then distribute them to the downstream protein sorting machineries (Ahting et al., 1999; Dudek et al., 2013; Bohnert et al., 2015; Fukasawa et al., 2017). It has been reported that the β-barrel protein TOM40 is the core subunit of TOM complex. Previous research about TOM40 focused on the biochemical characterization and its structure (Hill et al., 1998; Ahting et al., 2001; Gessmann et al., 2011; Bausewein et al., 2017). In yeast, TOM40 is a ∼40 kD pore protein that comprises a membrane-inserted, 19-stranded β-barrel domain with three α-helical domains at the N terminus and C terminus (Künkele et al., 1998; Hill et al., 1998; Lackey et al., 2014; Kuszak et al., 2015). These structures form the function area of TOM40 as the major entry gate for the mitochondrial preproteins. To characterize the properties of individual Tom40, single molecule tracking technique was utilized to trace individual Tom40 molecules in yeast, and the results demonstrated that Tom40 movement in the outer mitochondrial membrane is highly dynamic but confined in nature, suggesting anchoring of the TOM complex as a whole (Kuzmenko et al., 2011).

It is known that mitochondria play essential roles in the process of plant growth, development, and morphogenesis (Millar et al., 2008; Martin et al., 2014; Colas des Francs-Small and Small, 2014; Selles et al., 2018). Lately bioinformatic and genetic approaches were utilized in *Arabidopsis* to detect the genes involved in the process of germination, among the 239 genes queried 29 showed embryo arrested or lethal phenotypes (Law et al., 2012). The mitochondrial protein import component, translocase of the inner membrane17-1, was reported to play a role in defining the timing of germination in *Arabidopsis*. In mutants lacking *AtTIM17-1*, it showed a significant increase in the rate of germination, and altered mitochondrial biogenesis fed back to alter the germination rate (Wang et al., 2014). In *Arabidopsis*, a number of genes were identified to be mitochondrion-targeted and the mutation of these genes led to the aborted seeds at an early stage (Deng et al., 2014; Ju et al., 2016; Fromm et al., 2016). The CA domain is required for the complex I biogenesis in the mitochondrial respiratory chain. In *Arabidopsis*, mutants are affected in two specific CA subunits, and embryos show impaired mitochondrial membrane potential and mitochondrial reactive oxygen species (ROS) accumulation, and then trigger the lethal phenotype (Córdoba et al., 2016). Similar results were reported in maize (Gutiérrez-Marcos et al., 2007; Sosso et al., 2012; Ren et al., 2017), and rice (Xiao et al., 2018). It was considered that these mutations resulted in defective assembly of respiratory complexes in mitochondria, and then led to disorder in the mitochondria structure such as corruption in mitochondrial membrane, and subsequently caused reduced growth and fertility, or even embryo-lethality.

In plant, the TOM complex is generally composed of six subunits, they are TOM5, TOM6, TOM7, TOM9, TOM22, and TOM40. Previous research work demonstrated that TOM 5, 6, and 7 are small TOMs, they regulate the formation and stability of the TOM complex (Dietmeier et al., 1997; Kato and Mihara, 2008; Thornton et al., 2010; Becker et al., 2011). TOM9 presents as the counterpart of TOM22 in yeast, it is significantly smaller due to a truncation in the cytosolic receptor domain (Jänsch et al., 1998; Macasev et al., 2000; Parvin et al., 2017). TOM20 functions in receptor recognition (Werhahn et al., 2001; Lister et al., 2007), and TOM40 acts as the central porin of the TOM complex (Duncan et al., 2013a). A plant TOM complex was first purified from potato tuber mitochondria, and the sequence similarity between the 36-kDa protein and fungal TOM40 was identified (Jänsch et al., 1998). In *Arabidopsis*, through using biochemical and cell biology approaches, TOM40 was confirmed to be in the mitochondrial outer membrane and was a transmembrane β-barrel protein (Duncan et al., 2011). However, little was known about how TOM40 works in *Arabidopsis*. To explore the biological function of *AtTOM40* (At3g20000), a gene encoding the core subunit in the TOM complex of *Arabidopsis*, we obtained two independent T-DNA insertion lines (SALK_128170 and CS873594). With the aid of bioinformatic, genetic, molecular, cellular approaches, we characterize the comprehensive function of *AtTOM40*. Here we report that *AtTOM40* encodes a typical β-barrel protein and is conserved among different species, it is targeted to mitochondria and is widely expressed in different tissues. Knock-out of *AtTOM40* caused early embryo-lethal phenotype in *Arabidopsis*, which may be due to the failure of the mitochondrial biogenesis during embryo development. Further investigation revealed the expression of several important genes encoding outer mitochondrial membrane proteins and embryo pattern formation in *Arabidopsis* was disordered, suggesting that the AtTOM40 may participate in early embryo development through maintaining the normal mitochondrial biogenesis.

## Materials and Methods

### Plant Materials and Growth Conditions

*Arabidopsis thaliana* ecotype Columbia was used as materials. The two T-DNA insertion mutants SALK_128170 (*tom40-1/+*) and CS873594 (*tom40-2/+*) were obtained from the Arabidopsis Information Resource^1^. The *Arabidopsis* plants were cultivated in a greenhouse at Wuhan University at 22 ± 2°C with a 16-h light and 8-h dark photoperiod.

### Bioinformatic Analyses of TOM40 in Different Species

The AtTOM40 protein sequence was used to search for its homologs in other species by using BLAST^2^. Multiple sequence alignment was performed by ClustalX (version 1.83), and MEGA4 program was used to construct the phylogenetic tree based on the Neighbor-Joining algorithm (Tamura et al., 2007). A three dimensional structure of TOM40 in *Arabidopsis* and *Oryza sativa* were constructed according to the crystal structures of their yeast homologs through SWISS-MODEL^3^. Conserved function domains of TOM40 in different species were predicted on https://www.ebi.ac.uk/interpro/sequencesearch and then were demonstrated on http://ibs.biocuckoo.org/ website.

### Mutant Verification and Complementation Analysis

The *tom40-1/+* and *tom40-2/+* mutant lines were verified by using PCR with specific primers, respectively, and the insertion positions were confirmed by sequencing with the T-DNA primer. To complement the defects of *tom40-1/+* and *tom40-2/+*, the full-length genomic fragment of *AtTOM40* was amplified with KOD-Plus DNA polymerase (Toyobo) and cloned into *pCambia1300* vector (Cambia), and then transferred into the two mutants by using the floral dip method (Clough and Bent, 1998). Primers used in this work are listed in the Supplementary Table S1.

### Clearing Technique of Ovules and Observation of Endosperm Cellularization

Ovules from *Arabidopsis* were dissected from siliques with self-made needles and were cleared in Hoyer’s solution [chloral hydrate: glycerol: water, 8:1:2 (w/v/v)] for 30 min to 8 h depending on the different embryo developmental stages (Chen et al., 2015). To observe endosperm cellularization, ovules at different developing stages were conducted with the method described before (Li et al., 2017). All the specimens were observed under the Confocal Laser Scanning Microscope (CLSM, Leica SP8).

### RNA *in situ* Hybridization

Wild type ovule collecting, fixating, embedding, and sectioning, along with procedures of RNA *in situ* hybridization were performed as described previously (Brewer et al., 2006; Deng et al., 2014). The antisense and sense probes used in this study were all generated via PCR with T7 promoter adding primers (listed in Supplementary Table S1), and followed by *in vitro* transcription with the DIG RNA Labeling Kit (SP6/T7; Roche^4^). The ovule samples were observed under an Olympus BX60 microscope and photographed with the Olympus DP72 CCD.

### GUS Staining Analysis

The promoter fragment of *AtTOM40* was amplified by PCR, and its genome-specific primers are listed in Supplementary Table S1. After verification through sequencing, the amplified DNA fragment was cloned into *pCAMBIA1381Xb* (Cambia, Australia) vector, and then transformed into *Arabidopsis* wild type plants via the floral dip method described above. The homozygous *proAtTOM40::GUS* transgenic lines were screened and then used for GUS staining as described before (Chen et al., 2018). The stained samples were observed under an Olympus SZX12 microscope and photographed with a CCD (Cool SNAP, RS Photometric).

### Quantitative Real-Time PCR

Total RNAs from various tissues in *Arabidopsis* were extracted by using RNA isoPlus reagent (TaKaRa, Japan), and transcribed into cDNA with ReverTra Ace qPCR RT Kit (TOYOBO). The cDNAs were amplified as templates with the gene-specific primers, and quantitative Real-Time PCRs were carried out with a Rotor-Gene Q real-time PCR machine (Qiagen). The transcript abundance relative to WT of different tissues was analyzed by the comparative CT method as described previously (Schmittgen and Livak, 2008; Ma and Zhao, 2010), and the GLYCERALDEHYDE-3-PHOSPHATE DEHYDROGENASE (*GAPDH*) was utilized as an internal gene for normalizing (Zhong and Simons, 1999). Three independent biological replicates and three technical replicates of every sample were conducted for quantitative real-time PCR analysis. Primers used in the experiments are listed in Supplementary Table S1.

### Subcellular Localizations

The CDS (coding sequence) fragment of *AtTOM40* was amplified by PCR (the genome-specific primers are listed in Supplementary Table S1). After verification by sequencing, the amplified DNA fragment was cloned into pCAMBIA1300-eGFP vector, and then transformed into *Arabidopsis* wild type plants via the floral dip method described above. The homozygous *proAtTOM40*::CDS-GFP transformants were used for the subcellular localizations. Preparation and observation of the mesophyll protoplasts were conducted with the method described before (Deng et al., 2014). Mesophyll protoplasts isolated from the 4-week-old transformants were incubated with 2 mM MitoTracker Red FM (Molecular Probes) for 10 min and examined with a Confocal Laser Scanning Microscope (CLSM, Leica SP8). The images were taken under the eGFP fluorescence (excitation, 488 nm; emission, 505–530 nm) and the MitoTracker channel (excitation, 559 nm; emission, 530–560 nm).

### Transmission Electron Microscopy

The ovules at 6DAP (day after pollination) in wild type siliques and the albino ovules 6DAP in *tom40-1/+* siliques were collected for fixating, embedding and sectioning as described before (Ke et al., 2017). The ultrathin sections were cut with a Leica UC7 ultramicrotome, and observed under a transmission electron microscope (JEOL, JEM-1400 plus) and photographed with a JOEL digital camera EM (14800RUBY).

## Results

### AtTOM40 Is a Mitochondrial Translocase of the Outer Membrane and Is Highly Conserved Among Different Species

**FIGURE 1:**
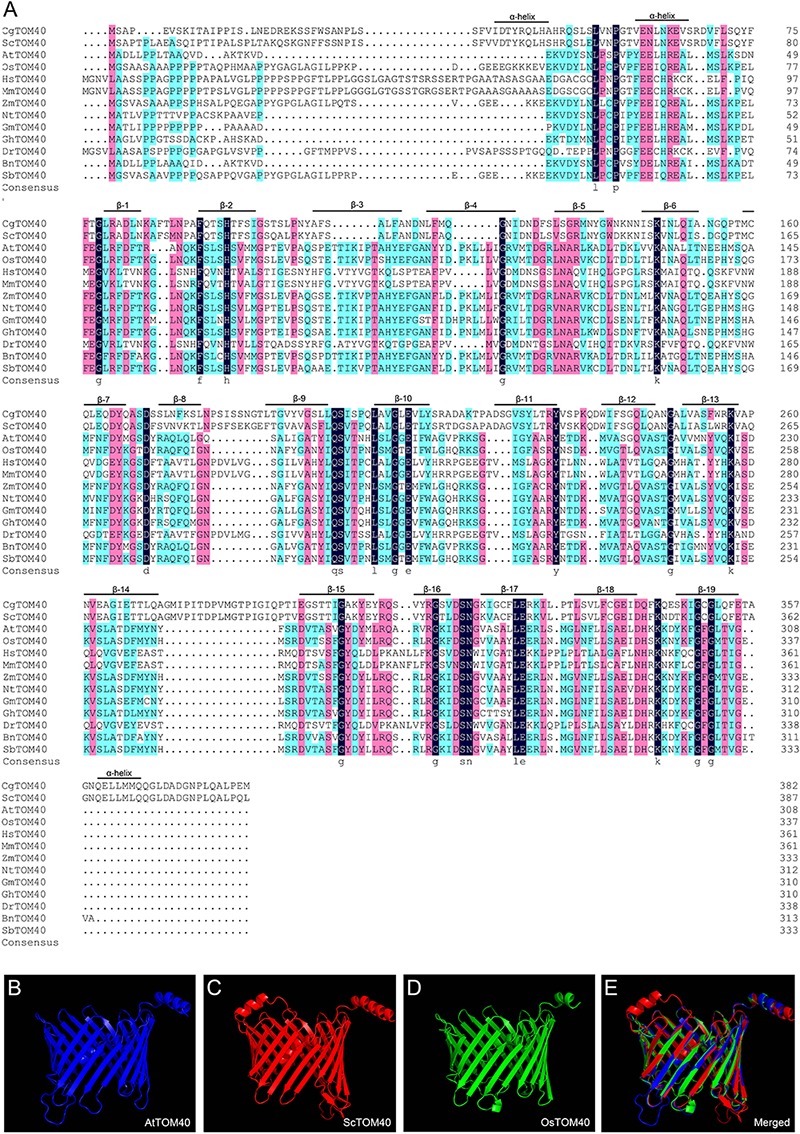
The conservation analysis of TOM40 in different species. **(A)** Sequence alignment of TOM40 in different species, α-helical segments and β-barrel domain were labeled according to *C. glabrata*. Cg, *Candida glabrata*; Sc, *Saccharomyces cerevisiae*; At, *Arabidopsis thaliana*; Os, *Oryza sativa*; Hs, *Homo sapiens*; Mm, *Mus musculus*; Zm, *Zea mays*; Nt, *Nicotiana tabacum*; Gm, *Glycine max*; Gh, *Gossypium hirsutum*; Dr, *Danio rerio*; Bn, *Brassica napus*; Sb, *Sorghum bicolor*. **(B–D)** Predicted 3-D structures of AtTOM40, ScTOM40 and OsTOM40 according to the crystal structures of their homologs in yeast. **(E)** The merged view of AtTOM40, ScTOM40 and OsTOM40 predicted 3-D structures.

To determine the identity of TOM40 among different species, we performed a full alignment of the amino acid sequences in AtTOM40 and its homologs from other species. The result showed that all the TOM40 proteins comprise 19 transmembrane β-barrel domains with two α-helical segments (Figure 1A), which are the conserved domains reported before (Kuszak et al., 2015). An extra α-helical segment was found only in *Saccharomyces* and *Candida glabrata*. The three-dimensional structure of TOM40 was constructed in *Arabidopsis thaliana*, *Saccharomyces cerevisiae* and *Oryza sativa*, the predicted 3D structure demonstrated the similar 19-stranded β-barrel, which forms the characteristic protein-conducting channel (Figure 1B–E). To further illustrate the evolutionary relationship among monocots, dicots, mammals, yeast and fungi, we analyzed the phylogenetic tree of TOM40s, and found a high degree of homology between AtTOM40 and other TOM40s (Supplementary Figures S1A,B). The conservation of TOM40s in sequence and structure indicates the protein as a common element in the mitochondrial outer membrane.

### Knock-Out of *AtTOM40* Causes Seed Abortion

**FIGURE 2:**
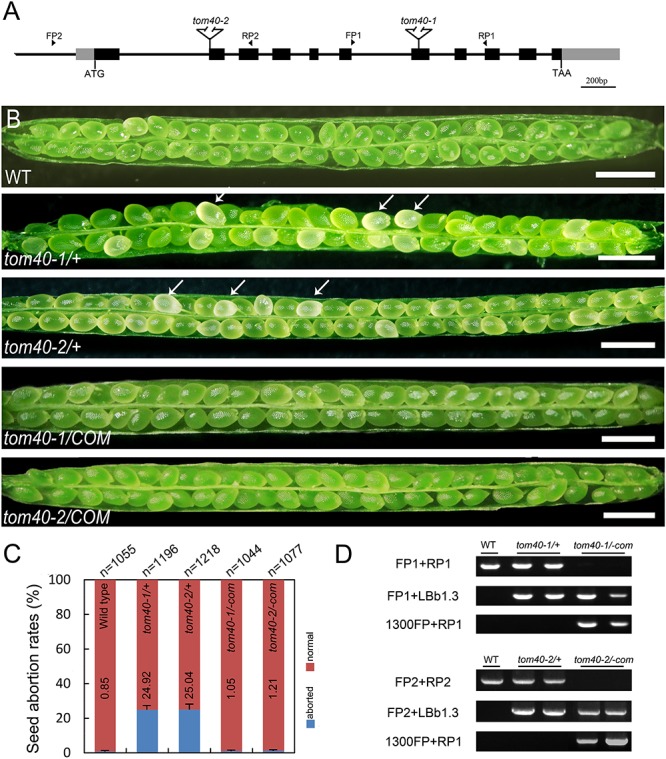
Characterization of *AtTOM40* mutation and complementation assays of *tom40-1/*+ and *tom40-2/+* mutants in *Arabidopsis*. **(A)** Schematic diagram of the *AtTOM40* gene and T-DNA positions of the two mutants. Exons and promoter are shown in black and gray boxes, and introns are shown as lines, respectively. FP and RP indicate forward and reverse primers, Bar = 200 bp. **(B)** Ovule phenotypes in siliques of the wild type, *tom40-1/+*, *tom40-2/+*, functionally complemented *tom40-1*/+ and *tom40-2/+* transgenic plants. The white arrows showed the aborted ovules. Bar = 1 mm. **(C)** Genotypic analysis of the wild type, *tom40-1/+*, *tom40-2/+* and the complemented mutant plants. **(D)** The seed abortion rates in the wild type, *tom40-1/+*, *tom40-2/+*, and the complemented mutant plants. The total numbers of seeds counted are listed on the top, and the seed abortion rates are shown on the bottom.

To investigate the function of TOM40 in *Arabidopsis*, we obtained two independent T-DNA insertion mutant lines SALK_128170 (*tom40-1*) and CS873594 (*tom40-2*) from the Arabidopsis Information Resource^5^. The positions of the T-DNA insertions in the *tom40-1* and *tom40-2* mutants were verified by genomic PCR and sequencing, and were found to be located in the seventh exon and the second exon, respectively (Figure 2A). Due to the loss of kanamycin resistance of the *tom40-1* mutant, we utilized PCR to analyze the genetic segregation of its self-fertilized progenies. The results showed that the selective ratio was about 2:1 (resistant: sensitive), instead of the expected 3:1, which suggested that the T-DNA insertion caused embryo lethality in recessive homozygous mutant plants. Moreover, the reciprocal crosses of mutant *tom40-1* with wild type plants showed that the segregation ratio was about 1:1 (resistant: sensitive), similar with expected ratio (Table 1), implying the gametophyte fertility of both females and males were normal. Similar results were obtained in the *tom40-2* mutant plant, and the segregation analysis of the self-fertilized mutant progenies was conducted with resistance screening (BASTA) instead. Furthermore, we dissected mature seeds from the wild type, *tom40-1/+* and *tom40-2/+* siliques, and found that the seeds in the wild-type can perform normal development, while both mutant plants possessed a proportion of aborted white seeds (Figure 2B). The seed abortion rates of two mutants were counted to be 24.92 and 25.04% respectively, which are close to the expected 25% (Figure 2C). The results demonstrated that the mutation of *AtTOM40* did not affect the gametophyte fertility, but could cause embryo lethality in homozygous progenies.

**Table 1:** Segregation ratios of the *tom40-1/+* and *tom40-2/+* mutants in *Arabidopsis*.

	With	Without
	T-DNA	T-DNA
	insert	insert		Ratio	Expected
Crosses (Female × Male)	(W)	(Wo)	Number	(W/Wo)	Ratio
*tom40-1*/+ × *tom40-1*/+	161	81	242	1.99:1	3:1
Col × *tom40-1/+*	70	73	143	0.96:1	1:1
*tom40-1*/+ × Col	71	72	143	0.99:1	1:1
*tom40-2*/+ × *tom40-2*/+	1822	898	2720	2.03:1	3:1
Col × *tom40-2/+*	342	337	679	1.01:1	1:1
*tom40-2/+* × Col	480	453	933	1.06:1	1:1


To confirm whether the seed lethality was caused by knock-out of the *AtTOM40* gene, we employed complementation tests. The full-length genomic sequence of *AtTOM40* was introduced into two mutants, and the self-progenies of T3 generation homozygous transgenic plants were analyzed by genotyping (Figure 2D). The results displayed that no aborted seeds were observed in both complementation lines (Figure 2B), and the ratio of the aborted seeds descended to the similar level with wild type (Figure 2C), indicating that the seed-lethal phenotype could be rescued by the introduction of *AtTOM40*. All the results support the conclusion that the knock-out of *AtTOM40* gene caused seed abortion, suggesting that the gene may play a critical role in embryo development in *Arabidopsis*.

### *AtTom40* Is Essential for Embryo Development and Pattern Formation

**FIGURE 3:**
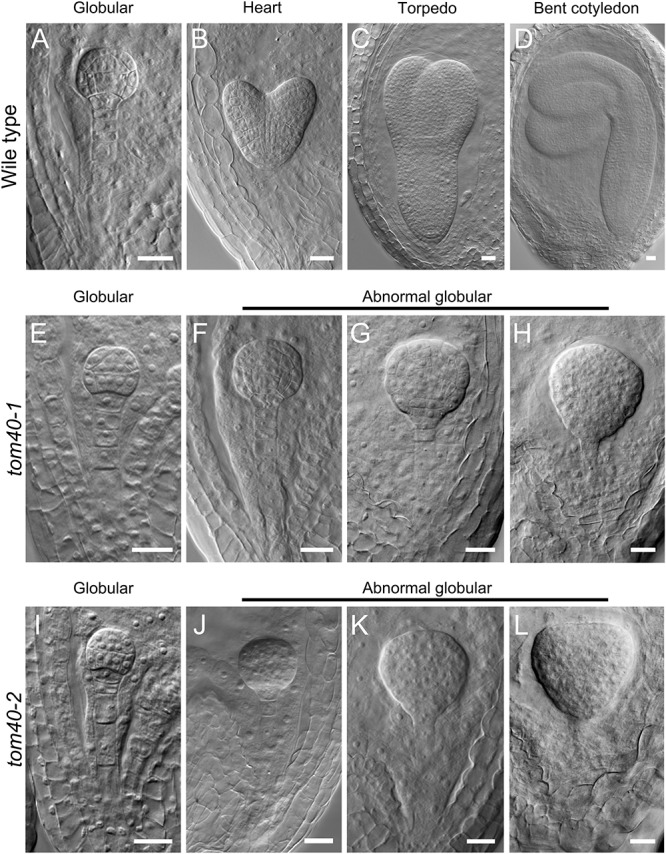
Status of embryo development in plants of the different genotypes. **(A–D)** Embryos from the globular stage to the bent cotyledon stage in wild-type ovules. **(E–H)** Embryos in homozygous *tom40-1* ovules at parallel developmental stages with **A–D**. **(I–L)** Embryos in homozygous *tom40-2* ovules at parallel developmental stages with **A–D**. Bar = 20 μm.

To clarify how the seed abortion occurred in the mutant plants, we monitored the process of embryo development with a whole mount clearing technique of ovules. The results showed that the difference was not noticeable between wild-type and *tom40-1* homozygous embryos before the late globular stage (Figure 3A,E). However, from the time that the wild-type embryos developed into the heart stage (Figure 3B), the embryos in the homozygous *tom40-1* mutant began to divide irregularly and could not produce proper organ primordium, including cotyledons and shoot apical meristem (SAM; Figure 3F). As development time goes, wild-type embryos progressed into torpedo and bent cotyledon stages (Figure 3C,D), while the abnormal embryos in the mutant plants still presented irregular globular embryos and could not produce cotyledon primordium (Figure 3G,H). In addition, despite the defect presented in embryo morphological development, the size of embryo propers in homozygous *tom40-1* mutant still expanded gradually as developmental time goes, but finally was trapped and arrested (Supplementary Figure S2A). The results indicated that the embryo development and pattern formation were affected in the homozygous mutant plants, and embryo lethality occurred eventually. Next, we calculated ratios of embryos at sequential development stages in wild type and heterozygote plants. The results demonstrated that the *tom40-1* mutant delayed growth as developmental progresses, and 24.39% of embryos at 6DAP were arrested at globular stages (Table 2). The frequency was consistent with the seed abortion rate of the mutant (Figure 2C), and similar results were obtained in *tom40-2* mutant plants (Figure 3I–L and Table 2), indicating that the proper function of the *AtTOM40* gene is required for the programmed embryogenesis in *Arabidopsis*.

**Table 2:** Ratios of *Arabidopsis* embryos in the wild type and the mutants at sequential development stages.

Genotype	Days after pollination	Sequential development stages (%)				
		
		Globular	Heart	Torpedo	Bent cotyledon	Number of seeds
Wild type	3	80.84	19.16	—	—	261
	4	9.92	90.08	—	—	242
	5	1.17	16.67	82.16	—	342
	6	—	1.36	8.81	89.83	295
*tom40-1*	3	87.94	12.06	—	—	315
	4	35.81	64.19	—	—	363
	5	23.50	11.16	65.34	—	251
	6	24.39	—	34.55	41.06	246
*tom40-2*	3	100	—	—	—	201
	4	25	75	—	—	248
	5	23.21	8.43	67.09	1.27	629
	6	24.83	4.59	13.78	56.80	588


Since the embryo and endosperm are both the major parts of seeds in *Arabidopsis*, we also examined the endosperm development status in wild type and *tom40-1* mutant through its autofluorescence. The results showed that the endosperm free nucleus initiated cellularization at the heart stage in wild type (Supplementary Figure S2B), and the initiation of endosperm cellularization was also observed in both the heterozygous and homozygous *tom40-1* mutant ovules at the same developmental stage (Supplementary Figures S2C,D). In brief, we draw the conclusion that *AtTOM40* is responsible for seed abortion in mutant plants, and the loss of it can affect embryo differentiation and pattern formation, but not endosperm free nuclear division and cellularization.

### *AtTOM40* Is Widely Expressed in Different Tissues and Especially in Cotyledons Primordium

**FIGURE 4:**
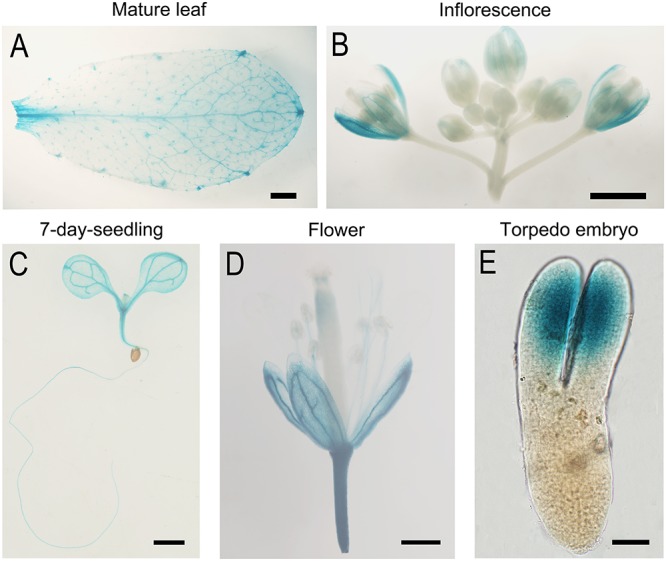
GUS Staining analysis of *TOM40* in *Arabidopsis*. **(A–E)** GUS staining signals (blue staining) in different tissues of *pTOM40::GUS* transgenic plants. **(A–D)** Bar = 1mm, **(E)** Bar = 20 μm.

To study the expression pattern of the *AtTOM40* gene, we obtained transgenic plants with *ProAtTOM40::GUS* construct. As a result, GUS signals could be detected in the veins of mature leaves (Figure 4A), sepals (Figure 4B), and 7-day-old seedlings (Figure 4C), respectively, especially in shoot meristems, hypocotyls, and vascular bundles of cotyledons (Figure 4C). However, the signal could be barely detected in anthers, stigmas and ovaries (Figure 4D), implying that the absence of *AtTOM40* might not affect gametophyte development, which was consistent with the results mentioned above (Table 1). Furthermore, we observed that GUS activity accumulated intensively in the cotyledon of the matured embryos (Figure 4E), hinting the important role of *AtTOM40* in cotyledon function.

**FIGURE 5:**
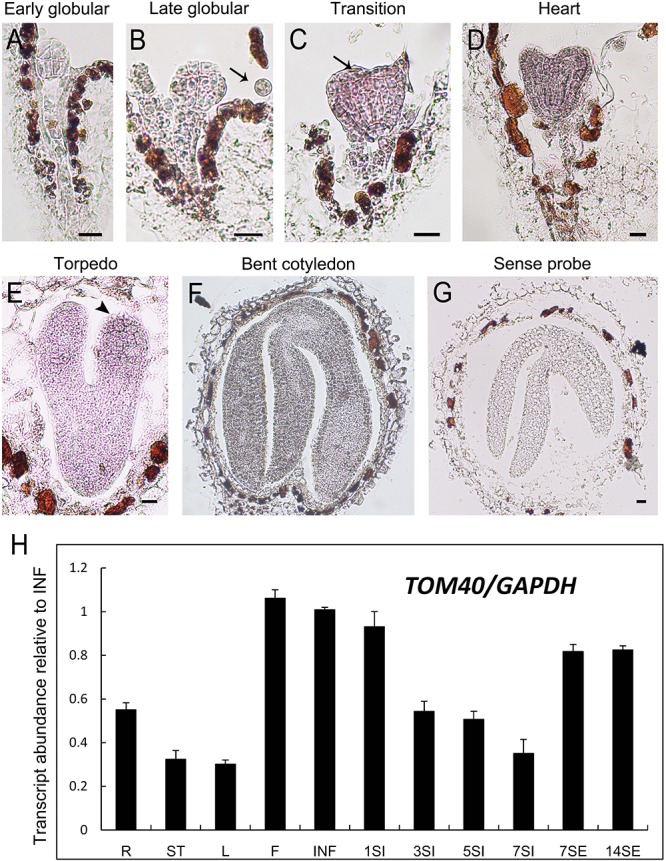
Expression analysis of *TOM40* in *Arabidopsis*. **(A–G)** *In situ* hybridization of *AtTOM40* transcripts (purple staining) accumulated in wild-type embryos and endosperm. Bar = 20 μm. **(H)** Quantitative real-time PCR analysis of *AtTOM40* in various tissues. R, root; S, stem; L, leaf; F, flower; INF, inflorescence; 1SI–7SI, siliques at 1, 3, 5, 7 days after pollination; 7-14SE, seedlings at 7 and 14 days after germination.

Through *in situ* hybridization technique, we detected the expression pattern of *AtTOM40* in wild type ovules. The results showed that the expression signals were present both in embryos and endosperms (Figure 5A–G, arrows), especially in cotyledons of embryos (Figure 4E, arrowhead), demonstrating the role of *AtTOM40* in *Arabidopsis* embryogenesis. To further investigate the expression pattern of the *AtTOM40* gene, we conducted quantitative real-time PCR to measure its expression in different tissues and organs. The results showed that *AtTOM40* gene is expressed extensively in almost all the organs at different levels, and is especially abundant in flowers (F), inflorescences (INF) and siliques at 1 day after pollination (Figure 5H). Taken together, we can draw the conclusion that *AtTOM40* is widely expressed in different tissues, especially in cotyledons primordium.

### AtTOM40 Is Localized in Mitochondria and Involved in Maintaining Normal Mitochondrial Biogenesis

**FIGURE 6:**
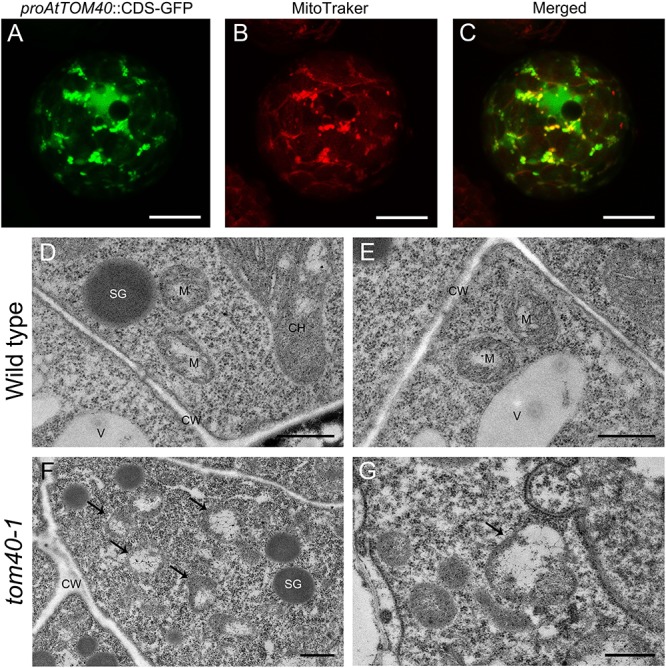
**(A–C)** Subcellular localization of AtTOM40 protein. Fluorescent signals in mesophyll protoplasts represents *AtTOM40* (green), mitochondria (red) and colocalization (merged), respectively. Bar = 10 μm. **(D–G)** Ultrastructure of mitochondria in wild type **(D,E)** and *tom40-1* **(F,G)** embryo proper cells at 6DAP. Bar = 500 nm. **(F,G)** Cells in *tom40-1* embryo proper at 6DAP. **(D,E,G)** Bar = 500 nm, **(F)** Bar = 1 μm. SG: starch grain; M: mitochondrion; CH: chloroplast; V: vacuole; CW: cell wall.

As predicted above, AtTOM40 is a typical mitochondria translocase of the outer membrane (Figure 1). To confirm this, we fused the *AtTOM40* promoter with enhanced GFP (eGFP) at the N-terminus to study the subcellular localization of AtTOM40 protein, in mesophyll protoplasts from the transgenic plants by confocal observation. As a result, the green fluorescence presents the localization of the AtTOM40 protein (Figure 6A), and the red fluorescence presents the localization of mitochondria after incubation with MitoTracker Red FM (Figure 6B). The green fluorescence and the red fluorescence were dotted displayed and could overlapped with each other (Figure 6C), indicating that the AtTOM40 localizes in mitochondria. To further investigate the ultrastructure of mitochondria in embryos, we collected ovules at 6DAP from *tom40-1* homozygous mutant and wild type siliques and observed them under TEM (transmission electron microscopy). The mitochondria in wild type embryo proper cells were well developed (Figure 6D,E), but was swollen with less intramitochondrial content and no regular cristae structure in the *tom40-1* mutant embryo proper cells (Figure 6F,G). The results demonstrate that AtTOM40 localizes in mitochondria and the lack of it impedes the proper biogenesis of mitochondria, suggesting the critical role of *AtTOM40* in maintaining mitochondrial function in *Arabidopsis*.

### Expression of Genes Encoding Outer Mitochondrial Membrane Proteins in the Homozygous *tom40-1* Mutant Embryos Were Affected

Previous research work has demonstrated that the multi-subunit TOM complex acts as an entry gate on the outer mitochondrial membrane (OMM), and recognizes mitochondrial precursor proteins and passes them through the outer membrane (Ahting et al., 1999; Dudek et al., 2013; Bohnert et al., 2015; Fukasawa et al., 2017). In *Arabidopsis*, there still exist a series of other proteins on the OMM. They function in import and assembly, transport, morphology and membrane dynamics, energy and metabolism, and synthetase related to the OMM (Duncan et al., 2013b). To further explore how the mutation of *AtTOM40* influenced the proteins on OMM, we performed quantitative real-time PCR to determine the changes of expression levels of OMM protein-related genes.

**FIGURE 7:**
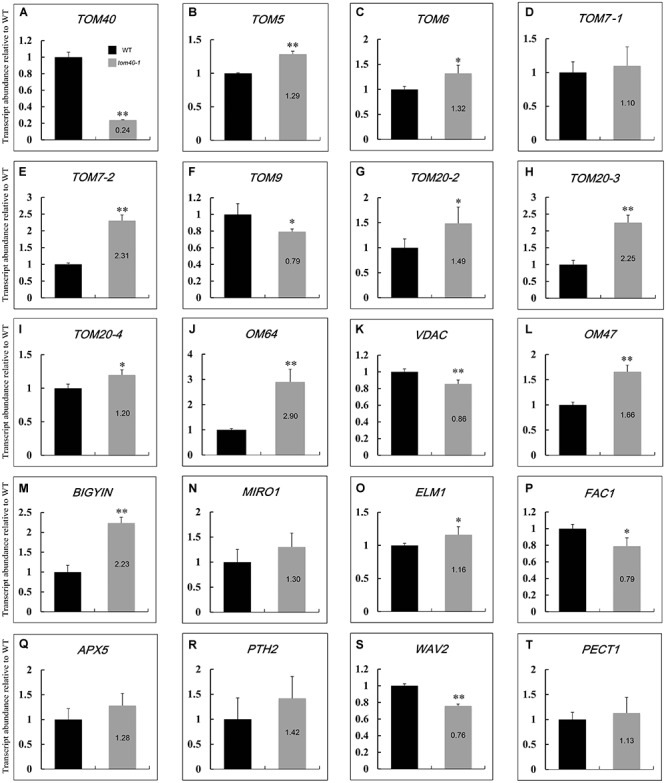
Relative expression levels of genes relevant to outer mitochondrial membrane (OMM) in *Arabidopsis* between wild type and *tom40-1* albino ovules at 6DAP. **(A)** Expression level of *AtTOM40* in the *tom40-1* albino ovules was about one fifth of the expression level in the wild type. **(B–J)** Expression levels of genes involved in import and assembly of mitochondrion proteins. **(K–L)** Expression levels of genes involved in protein transport in mitochondrion. **(M–O)** Expression levels of genes involved in morphology and membrane dynamics. **(P–Q)** Expression levels of genes involved in energy and metabolism. **(R–T)** Expression levels of genes involved in synthetase related to the OMM. The asterisk indicates a significant difference (Student’s *t*-test, ^∗^*P* < 0.05, ^∗∗^*P* < 0.01).

Considering that the albino ovules in *tom40-1/+* plants were obvious at 6 days after pollination, we collected 6 DAP ovules in wild type and 6 DAP albino ovules in *tom40-1/+* plants respectively for quantitative real-time PCR. At this time, embryos in the wild type had developed to the bent cotyledon stage (Figure 3D), while the abnormal embryos in *tom40-1* plants were arrested at the irregular globular stage (Figure 3H). The preliminary experiment showed that the expression level of *AtTOM40* in the *tom40-1* albino ovules was about one fifth of the expression level in the wild type (Figure 7A), hence the subsequent experiments were reliable and feasible. The expression data showed that among 19 genes tested about three quarters genes were significantly up-regulated or down-regulated (Figure 7B–T). Among the genes involved in import and assembly (Figure 7B–J), transport (Figure 7K–L), morphology and membrane dynamics (Figure 7M–O), most genes were significantly up-regulated, which may due to the absence of *AtTOM40* gene. In the meantime, among the genes that function in energy and metabolism (Figure 7P–Q), and synthetase related to the OMM (Figure 7R–T), about a half of the genes were down-regulated, which meant not all the OMM proteins were affected at the transcriptional level. In brief, the mutation of the *AtTOM40* gene led to the abnormal expression of genes encoding OMM proteins in *Arabidopsis*.

### Expression of Genes Relevant to Embryo Development and Pattern Formation in Homozygous *tom40-1* Mutant Embryos Were Abnormal

It has been reported that the embryo development and pattern formation in *Arabidopsis* are precise and predictable. A complicated network control the specification of primordium of various tissue, and a number of genes have been proved to be involved in this process (Tzafrir et al., 2004; ten Hove et al., 2015; Palovaara et al., 2017). In this study, we found that mutation of *AtTOM40* led to abnormal embryo development through affecting its pattern formation, and the embryos showed shape alterations and were unable to produce proper tissue primordium, including cotyledon and the shoot apical meristem (SAM; Figure 3). To further evaluate the effects of *AtTOM40* mutation on embryo morphological development, we performed quantitative real-time PCR to determine the changes of expression levels of genes known to be essential for embryo pattern formation. The experiment was conducted in the 6 DAP ovules of wild type and 6 DAP albino ovules of *tom40-1/+* siliques, respectively.

**FIGURE 8:**
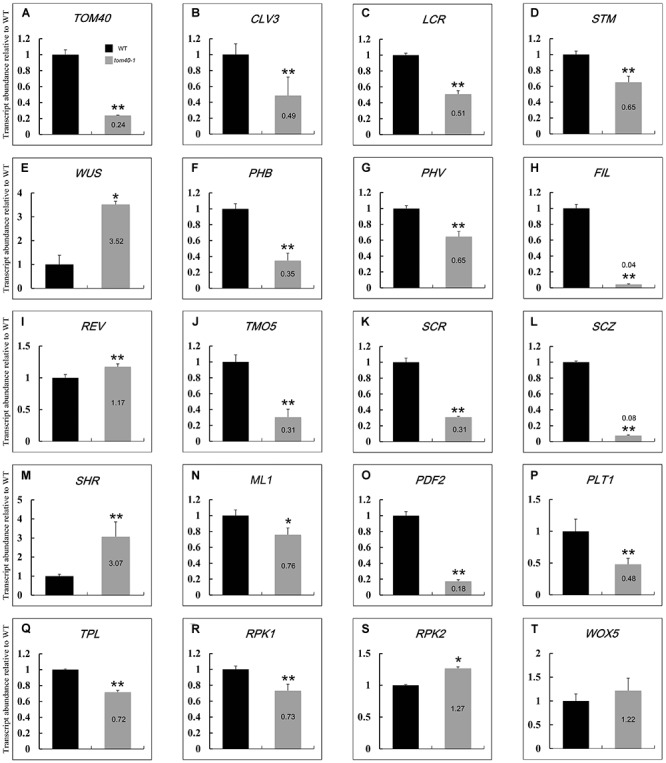
Relative expression levels of genes involved in embryo cell fate decision between wild type and the *tom40-1* albino ovules at 6DAP. **(A)** Expression level of *AtTOM40* in the *tom40-1* albino ovules was about one fifth of the expression level in the wild type. **(B–E)** Expression levels of genes involved in SAM. **(F–I)** Expression levels of genes involved in cotyledon. **(J–M)** Expression levels of genes involved in vascular primordium. **(N,O)** Expression levels of genes involved in protoderm. **(P–T)** Expression levels of genes involved in hypophysis. The asterisk indicates a significant difference (Student’s *t*-test, ^∗^*P* < 0.05, ^∗∗^*P* < 0.01).

As a result, of the 19 genes tested, 18 of them were significantly abnormally expressed. The 18 abnormally expressed genes are consistent with the phenotype indicating major defects in patterning of the embryo, demonstrating that the embryo patterning was severely affected at the transcriptional level in the homozygous embryos of *tom40-1/+* plants. Except for the *WUS* (*WUSCHEL*) gene, the genes expressed in the SAM, including *CLV3* (*CLAVATA3*), *LCR* (*LEAF CURLING RESPONSIVENESS*), and *STM* (*SHOOT MERISTEMLESS*), were significantly down-regulated, (Figure 8B–E), which was correspondent with the abnormal shoot apical meristem development in the homozygous *tom40-1* embryos (Figure 3F–H). Similar results were obtained in genes expressed in cotyledon (Figure 8F–I) and vascular primordium (Figure 8J–M). In addition, two genes expressed in protoderm (*ML1*, *MERISTEM LAYER 1* and *PDF2*, *PROTODERMAL FACTOR 2*) were significantly down-regulated (Figure 8N,O), indicating the severe disorder of their expression in the protoderm. Furthermore, among the 5 regulators of hypophysis, 4 of them were significantly abnormally expressed (Figure 8P–S) except that the expression level of WOX5 (*WUSCHEL RELATED HOMEOBOX 5*) was almost unaffected (Figure 8T). In conclusion, the embryonic pattern formation was abnormal at transcriptional level in the homozygous *tom40-1* mutant, indicating that the *AtTOM40* gene plays an essential role in the embryo development and pattern formation in *Arabidopsis*.

## Discussion

### TOM40 Is Essential for the Mitochondria Biogenesis in Different Species

Mitochondria are ubiquitous organelles given rise from endosymbiotic proteobacteria during evolution, and bounded by double-layer membranes (Dolezal et al., 2006; Bohnert et al., 2015). In the course of evolution, copies of genes were transferred from the mitochondrial DNA to the nuclear genome, and vast majority of mitochondrial proteins are synthesized in the cytosol (Perry et al., 2008). Preproteins synthesized in the cytosol are imported and translocated into the mitochondria, and the import process was mediated by membrane protein complexes named translocase. The mitochondrial translocase of the outer mitochondrial membrane (TOM) complex plays important role as an entry gate for nuclear-encoded mitochondrial precursor proteins (Vestweber et al., 1989; Bohnert et al., 2015), and TOM40, a central porin, serves as the core subunit in the complex (Duncan et al., 2013a; Murcha et al., 2014). Previous research demonstrated that TOM40 is a component of the mitochondrial protein import site, and forms the hydrophilic channel of the mitochondrial import pore for preproteins (Perry et al., 2008; Lackey et al., 2014). In yeast, it has been reported that Tom40 functions as the direct binding partner of Vps39 on mitochondria, therefore *Vps39* mutant was defective in TOM binding and has lower survival rate during starvation (González Montoro et al., 2018). Furthermore, it was also reported in yeast that the levels of O^2-^ released from the mitochondria coincide with TOM40 expression level. When blocking the TOM complex, it could result in inhibition of O^2-^ release from the mitochondria, indicating the role of TOM40 in respiration of mitochondria (Budzińska et al., 2009). In this study, we found that the *TOM40* gene in *Arabidopsis* was wildly expressed in different tissues and especially in cotyledons primordium (Figure 4, 5), it was also found to be localized in mitochondria and proper mitochondrial biogenesis was disturbed in homozygous *tom40-1* mutant embryos (Figure 6), implying that the knock-out of *AtTOM40* led to impaired mitochondria. This may be caused by the failure of the preprotein import.

In *Arabidopsis*, the outer mitochondrial membrane (OMM) proteins function in a series of biological events of mitochondria. TOM9, the plant homolog of yeast TOM22, was reported to be critical for TOM complex assembly, and the Dexamethasone induced RNAi gene silencing of TOM9.2 (the major isoform of TOM9) resulted in a severe reduction in the mature TOM complex (Parvin et al., 2017). Another plant-specific OMM protein AtOM47 was also reported, it plays a role in leaf senescence by transporting metabolic intermediates into and out of mitochondria (Li et al., 2016). In our study, the expression of genes encoding OMM proteins was further detected in the mutant *tom40-1* ovules (Figure 7). For the genes encoding the subunits of TOM complex, nearly all the expression level was significantly up-regulated, except that the expression of TOM9 was significantly down- regulated. The results implied that the stability of the TOM complex was disturbed due to the lack of AtTOM40. Moreover, the expression of other OMM proteins, including proteins involved in import and assembly, transport, morphology and membrane dynamics of mitochondria, was also disturbed in the *tom40-1* mutant, which strongly implied that *AtTOM40* gene may be crucial for normal expression of the OMM genes. Hence, we speculate reasonably that the lack of *AtTOM40* gene caused abnormity of the whole TOM complex and then disordered the mitochondrial import apparatus. Thus AtTOM40 is essential for the mitochondria biogenesis.

### TOM40 Plays an Important Role in Embryo Development and Pattern Formation Through Maintaining the Function of the Mitochondria Outer Membrane

Previous research has shown that mitochondria played a fundamental role in embryo development in plants (Colas des Francs-Small and Small, 2014), a number of genes located in mitochondria have been found to be essential for embryo development from the early stage in *Arabidopsis* (Yamaoka et al., 2011; Mansilla et al., 2015), and even caused seed abortion in *Arabidopsis* (Song and Liu, 2015; Guan et al., 2015; Fromm et al., 2016) and Maize (Wang et al., 2017). In *Arabidopsis*, the late embryogenesis abundant (LEA) proteins protect the mitochondrial inner membrane in the case of desiccation tolerance, in order to ensure the function of the energy-generating system. And the embryonal mitochondria undergo a process of development before becoming fully functional. Efficient import machinery are needed to allow proper biogenesis of mitochondria (Tolleter et al., 2007). It has also been reported that in rice and *Arabidopsis*, during the process of embryo development and seed maturation, appropriate preprotein import and mitochondrial biogenesis are necessary for bioenergetic and metabolic functions of mitochondria, including TCA cycle and electron transport chain assembly (Howell et al., 2006; Law et al., 2012). The *ETHE1* gene catalyzes the oxidation of persulfides in the mitochondrial matrix and is essential for early embryo development in *Arabidopsis* (Krüßel et al., 2014). The results in our lab showed that translocase of the inner membrane 9 and 10 (TIM9 and TIM 10) are essential for maintaining mitochondrial function during early embryo cell and endosperm free nucleus divisions in *Arabidopsis* (Deng et al., 2014). Furthermore, a series of evidence demonstrated that the morphogenesis was crucial for the production of embryo in plant development, and the molecular mechanism of the process was well verified (Tzafrir et al., 2004; Lau et al., 2012; ten Hove et al., 2015). In this study we confirmed that AtTOM40 was a typical translocase of the outer membrane, abnormal embryo development and seed abortion in homozygous *tom40* mutant embryos were caused by the absence of *AtTOM40* (Figure 2). We also found that embryo development and organ differentiation was suppressed in homozygous mutant embryos (Figure 3), and the failure of morphogenesis was correlated with the misexpression of tissue-specific genes (Figure 8). Taken together, our results revealed that *AtTOM40* was essential for the maintenance of normal mitochondrial biogenesis in *Arabidopsis*, and played an important role in the process of embryo development.

The mechanism of how is *TOM40* is involved in embryo development and morphogenesis remains unclear. It is known that from the globular stage to the heart and the later stage, a large number of cellular proliferation and organ primordial differentiation occurred in *Arabidopsis* embryos (Yoshida et al., 2014). These events laid the foundation for the mature embryo formation and the whole plant development, for which abundant energy productions are needed (Guitton et al., 2004; Xiang et al., 2011). As energy-supply organelles, mitochondria need proper biogenesis and efficient import machinery to provide sufficient ATP for the biological events. Hence, we speculate reasonably that the *AtTOM40* gene was involved in the quality control of mitochondria, and the proper function was essential for the biogenesis of mitochondria activities and structure. Therefore, the loss of *AtTOM40* led to the functional defect of mitochondria and affected the energy metabolism, and consequently caused the insufficient ATP supply for cell differentiation and embryo organ primordial development, and finally caused embryo lethality. Since mitochondria are maternally inherited in higher plants (Baroux et al., 2008; Nagata, 2010; Xin et al., 2012), it could be derived that in the mutants, the residual maternal energy could enable gametes to complete fertilization and undergo a few rounds of cell division after it. However, the mitochondrial defection may not be able to support the further cell differentiation and embryo organ primordial formation. Our results confirmed that the *AtTOM40* localized to the mitochondria and the loss of it would lead to impaired mitochondria structure and embryonic defection. It has been reported that the endosperm undergoes eight rounds of mitosis, and then was followed by cellularization. In the meantime, the embryo development had just reached the dermatogen stage (Boisnard-Lorig et al., 2001; Berger, 2003). Therefore, we can speculate reasonably that in the *tom40-1* mutant, the residual maternal energy could support only about eight rounds of cell divisions. Hence, the process of endosperm cellularization could be finished, while the embryo was arrested at the globular stage. Besides, the unparallel development may be derived from a signaling rather than metabolic defect (Sosso et al., 2012). Our work provides a novel insight about the biological function of TOM40 in *Arabidopsis*, and could help researchers to further understand the relationship between mitochondrial biogenesis and embryo development, but the exact mechanism of AtTOM40 still needs more detailed analyses and further investigation.

## Data Availability

All datasets generated for this study are included in the manuscript and/or the Supplementary Files.

## Author Contributions

YiH performed most of the experiments, analyzed the research results, and wrote the manuscript. WZ performed the ultrathin section and TEM observation. ZW and YuH performed the Quantitative Real-Time PCR. YZ and JQ participated in the RNA *in situ* hybridization. XW and YR participated in the mutant verification and complementation analysis. JZ conceived the research plans, guided the whole study, and modified the manuscript.

## Conflict of Interest Statement

The authors declare that the research was conducted in the absence of any commercial or financial relationships that could be construed as a potential conflict of interest.

## Footnotes

1 https://www.arabidopsis.org

2 https://blast.ncbi.nlm.nih.gov/Blast.cgi

3 https://www.swissmodel.expasy.org/

4 https://www.roche.com

5 https://www.arabidopsis.org

## Supplementary Material

Figure S1 The evolutionary relationship and conservation of TOM40 among different species. **(A)** Phylogenetic tree of TOM40 among different species. **(B)** Analyses of conserved domain in TOM40. Gray area represent the whole protein sequence and the blue area represents the conserved porin domain. Cg, *Candida glabrata*; Sc, *Saccharomyces cerevisiae*; At, *Arabidopsis thaliana*; Os, *Oryza sativa*; Hs, *Homo sapiens*; Mm, *Mus musculus*; Zm, *Zea mays*; Nt, *Nicotiana tabacum*; Gm, *Glycine max*; Gh, *Gossypium hirsutum*; Dr, *Danio rerio*; Bn, *Brassica napus*; Sb, *Sorghum bicolor.*

Figure S2 Developmental status of embryos and endosperm in plants of different genotypes. **(A)** Size of homozygous embryo propers in *tom40-1* and *tom40-2* at 3, 5, 7, 9 days after pollination (DAP). **(B–D)** The process of endosperm cellularization had initiated in wild type **(B)**, heterozygous **(C)** and homozygous **(D)** ovules at 4DAP. Bar = 20 μm.

Table S1 Primers (5′ to 3′) used in this study.
