# Supplementary figures and images for: Translocase of the Outer Mitochondrial Membrane 40 Is Required for Mitochondrial Biogenesis and Embryo Development in Arabidopsis
Source: Front Plant Sci. 2019 Apr 2;10:389. doi: 10.3389/fpls.2019.00389 (PMC6455079; doi:10.3389/fpls.2019.00389)

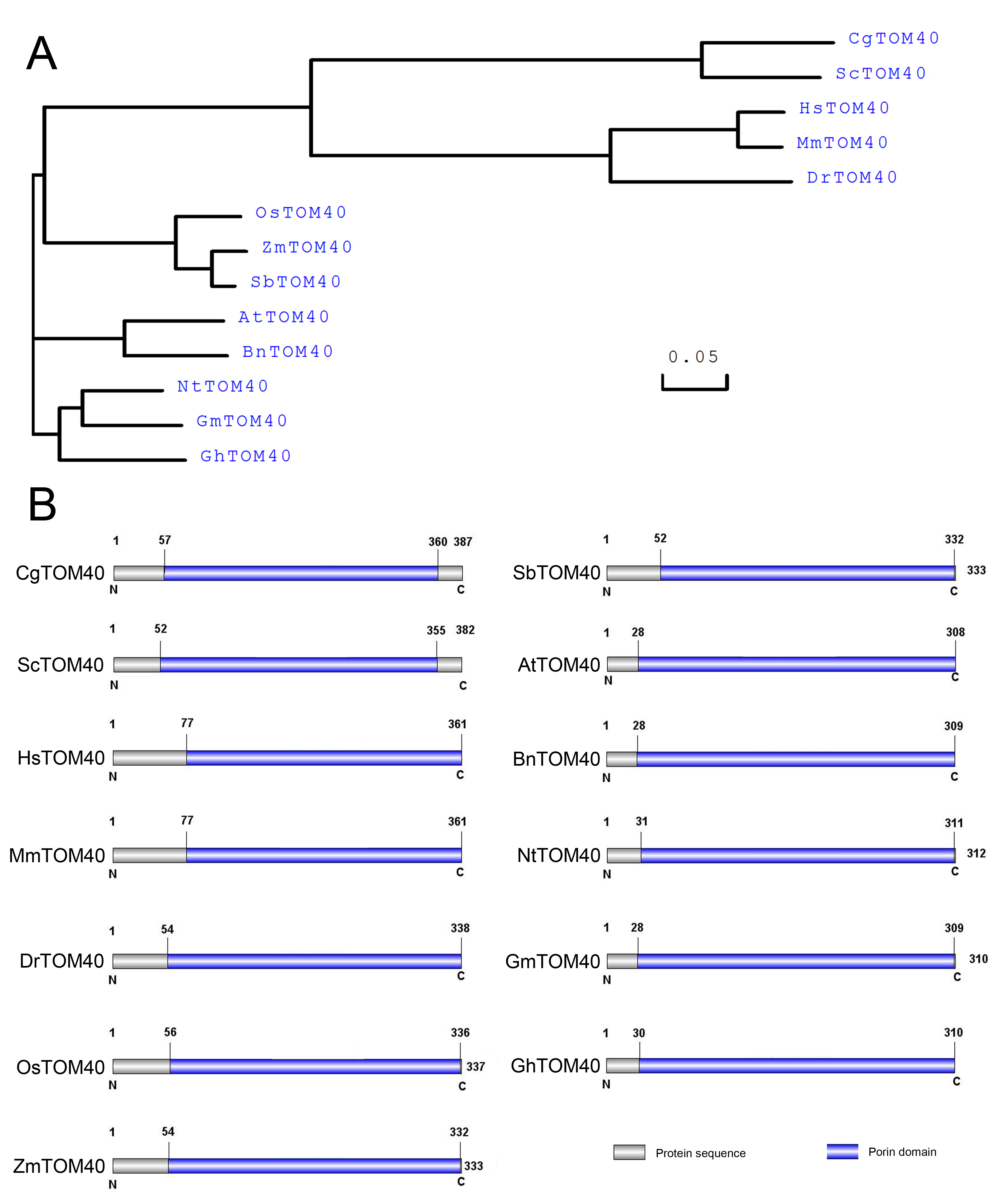

Supplement: Figure S1 — The evolutionary relationship and conservation of TOM40 among different species. (A) Phylogenetic tree of TOM40 among different species. (B) Analyses of conserved domain in TOM40. Gray area represent the whole protein sequence and the blue area represents the conserved porin domain. Cg, Candida glabrata; Sc, Saccharomyces cerevisiae; At, Arabidopsis thaliana; Os, Oryza sativa; Hs, Homo sapiens; Mm, Mus musculus; Zm, Zea mays; Nt, Nicotiana tabacum; Gm, Glycine max; Gh, Gossypium hirsutum; Dr, Danio rerio; Bn, Brassica napus; Sb, Sorghum bicolor. [file Image_1.JPEG]

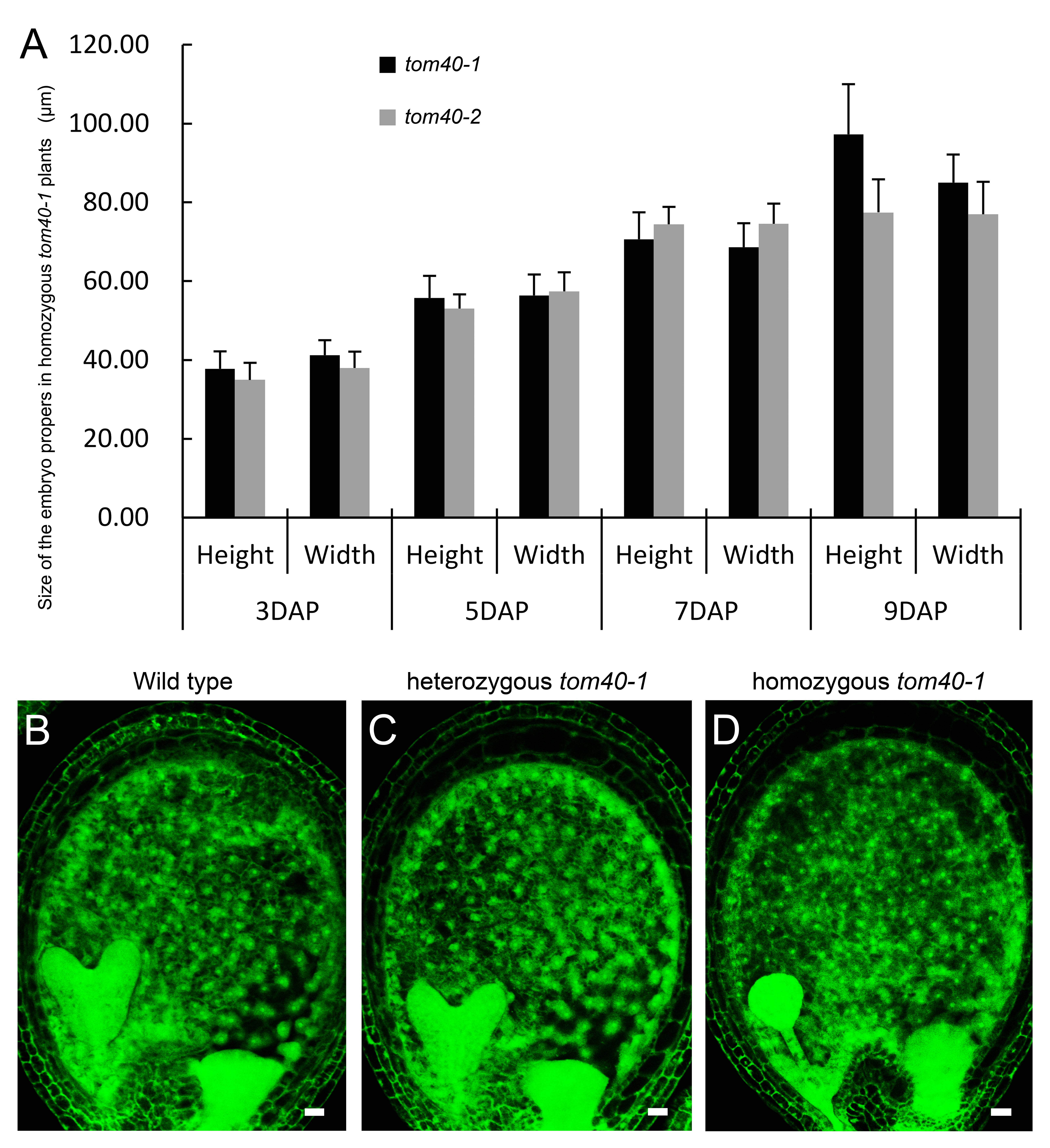

Supplement: Figure S2 — Developmental status of embryos and endosperm in plants of different genotypes. (A) Size of homozygous embryo propers in tom40-1 and tom40-2 at 3, 5, 7, 9 days after pollination (DAP). (B–D) The process of endosperm cellularization had initiated in wild type (B), heterozygous (C) and homozygous (D) ovules at 4DAP. Bar = 20 μm. [file Image_2.JPEG]
