# Supplementary material for: Translocase of the Outer Mitochondrial Membrane 40 Is Required for Mitochondrial Biogenesis and Embryo Development in Arabidopsis
Source: Front Plant Sci. 2019 Apr 2;10:389. doi: 10.3389/fpls.2019.00389 (PMC6455079; doi:10.3389/fpls.2019.00389)
Supplement: Table S1 — Primers (5′ to 3′) used in this study. [file Table_1.doc]

**Table S1 Primers (5' to 3') used in this study**

| **1.1 Primers for mutant verification** | | |
| --- | --- | --- |
|  | **Forward Primer** | **Reverse Primer** |
| *tom40-1* | gttagttgtgaaggcgaatgc | aaatcagtggcaagtgacacc |
| *tom40-2* | tttagccgcttaagaggttgg | taatgggctgttggaattttg |
| LBb1.3 | attttgccgatttcggaac | - |
| PAC161 | atattgaccatcatactcattgc |  |
| **1.2 Primers for complementation** | | |
| TOM40-Com | actaagcttaacttgggtctcgtgcgctaataa | actggatccccacgtcaaaagcaacaaatctca |
| PC1300 | cgggcctcttcgctattacg | aggcaccccaggctttacact |
| TOM40-1-Com identification | tgtttgccttgattcgtc | atgcgggattcagaaatg |
| TOM40-2-Com identification | tatcttgaacttgggtct | cgtaatttccagctacac |
| **1.3 Primers for GUS/GFP fusion constructs** | | |
| *TOM40*-Pro-GUS | actaagctttcggtcactttttaagagtctt | actagatcttccatcgccgcctctaccgctttt |
| *TOM40*-Pro-GFP | acttctagaatggcggatcttttaccacc | actggatccttaaccaactgttaatccga |
| **1.4 Primers for *in Situ* hybridization** | | |
| TOM40-ISH-S | atcctgcatctcaaatcatcatcccttctcctgttcccta | ctgattcaacgccctggta |
| TOM40-ISH-AS | tcccttctcctgttcccta | cataatacgactcactatagggctgattcaacgccctggta |

| **1.5 Primers for quantitive RT-PCR** | | | | |
| --- | --- | --- | --- | --- |
| Gene | Full Name | Isoform | Forward Primer | Reverse Primer |
| *GAPDH* | *GLYCERALDEHYDE-3-PHOSPHATE DEHYDROGENASE* | AT1G16300 | gagtctactggtgtcttcactg | caaggtcggacttgtattcgtg |
| *TOM40* | *TRANSLOCASE of the OUTER MEMBRANE 40* | AT3G20000 | actttaccagggcgttga | attacccttccgatgagc |
| *TOM5* | *TRANSLOCASE of the OUTER MEMBRANE 5* | AT5G08040 | tatcgaaaagatgaaagca | catgagatccccatagct |
| *TOM6* | *TRANSLOCASE of the OUTER MEMBRANE 6* | AT1G49410 | atgttcccaggaatgttc | agaaatcgatcttgagct |
| *TOM7-1* | *TRANSLOCASE of the OUTER MEMBRANE 7-1* | AT5G41685 | gcaaaggatcaaagggag | cagacagggctaaggagc |
| *TOM7-2* | *TRANSLOCASE of the OUTER MEMBRANE 7-2* | AT1G64220 | taaaagtacgttgaagatca | gagggatgaaaccatagtga |
| *TOM9* | *TRANSLOCASE of the OUTER MEMBRANE 9* | AT5G43970 | cgtcgaagtatcgaagaaa | gcattggagatggtggag |
| *TOM 20-2* | *TRANSLOCASE of the OUTER MEMBRANE 20-2* | AT1G27390 | gaacatgctcgcaaaaattctg | tggatttattgtcaaggcctct |
| *TOM 20-3* | *TRANSLOCASE of the OUTER MEMBRANE 20-3* | AT3G27080 | ggtgtattgggaatgcatacac | catccacagcttgttgaaagaa |
| *TOM 20-4* | *TRANSLOCASE of the OUTER MEMBRANE 20-4* | AT5G40930 | agccaaagcagatgattctaga | atgatttccgatagagttcgct |
| *OM64* | *OUTER MEMBRANE 64* | AT5G09420 | aatggaataaggcggtaa | agcagtccctcgtctcaa |
| *VDAC* | *VOLTAGE DEPENDENT ANION CHANNEL* | AT3G01280 | gacttaattgcctcccttac | gacacgagccttcacaga |
| *OM47* | *OUTER MEMBRANE PROTEIN 47* | AT3G27930 | cattcaagtcgtggtgga | tgttcggtgtcagcatta |
| *BIGYIN* | *TETRATRICOPEPTIDE REPEAT (TPR)-LIKE SUPERFAMILY PROTEIN* | AT3G57090 | cttcttgctgttgggtatt | aaaggctgtagccgtgat |
| *MIRO1* | *MITOCHONDRIAL RHO TYPE GTPASE 1* | AT5G27540 | aagatagggacattgttgctga | ggaagccagtattcactcaaac |
| *ELM1* | *ELONGATED MITOCHONDRIA 1* | AT5G22350 | tgttggagccgatcactg | acgccgtattctcgtagc |
| *FAC1* | *EMBRYONIC FACTOR 1* | AT2G38280 | ccagctcaatggactaac | aagcacaggagactttcg |
| *APX5* | *ASCORBATE PEROXIDASE 5* | AT4G35970 | gagtctcttacgcagaccttta | cccatacgagagaagagagttc |
| *PTH2* | *PEPTIDYL-tRNA HYDROLASE 2* | AT3G03010 | acatcagcactttacgacaaac | gttttcttattcccgctggatc |
| *WAV2* | *WAVY 2* | AT5G20520 | tggtttattggaggaagtg | ctttcgcatacagcattt |
| *PECT1* | *PHOSPHATIDYL ETHANOLAMINE CYTIDYLYL TRANSFERASE 1* | AT2G38670 | ggaagttgtatagtcggaggag | tggctataatttcttcgtcgga |
| *CLV3* | *CLAVATA3* | AT2G27250 | aagacagccaagaaacaa | cttaccaaacgaaacaga |
| *LCR* | *LEAF CURLING RESPONSIVENESS* | AT1G27340 | ctctgggaaatggaaaca | tcatctgtctggacctcaa |
| *STM* | *SHOOT MERISTEMLESS* | AT1G62360 | tagcctcgccacaacctc | ccttgctcctcttcctcttct |
| *WUS* | *WUSCHEL* | AT2G17950 | aaccaagaccatcatctctatcatc | tcagtacctgagcttgcatga |
| *PHB* | *PHABULOSA* | AT2G34710 | gcttgacgtgtggatccttct | cctttgcttccttccggtttc |
| *PHV* | *PHAVOLUTA* | AT1G30490 | cagcagaataggcatcgacac | tcctctgtgccaaagcttcta |
| *FIL* | *FILAMENTOUS FLOWE* | AT2G45190 | cggtgcatctctcagctcctc | tgtccgatgtggttgctgtac |
| *REV* | *REVOLUTA* | AT5G60690 | cttgtctgcgaaaatggatat | ccagcaggactattcgcatct |
| *TMO5* | *TARGET of MONOPTEROS 5* | AT3G25710 | aagaagacgagagagaatca | tgtgatgtttgtcgttttag |
| *SCR* | *SCARECROW* | AT3G54220 | ctggcttcaacattctcttta | tgcttctacaaatcttcctaa |
| *SCZ* | *SCHIZORIZA* | AT1G46264 | aaacataacaacttctctag | tctcctctcttgaaaaactc |
| *SHR* | *SHORT ROOT* | AT4G37650 | tagccacaagatcagacgac | atccgatgcgacgccgtttg |
| *ML1* | *MERISTEM LAYER 1* | AT4G21750 | aacggaagagatgctaaagct | aacgttgccgacctattctc |
| *PDF2* | *PROTODERMAL FACTOR 2* | AT4G04890 | ctagcttcaacatactcttc | ggctacactcgaacgacaat |
| *PLT1* | *PLETHORA 1* | AT3G20840 | gcattggacactttcggaca | ctggccttcccttctacaac |
| *TPL* | *TOPLESS* | AT1G15750 | gcgatgatattgttgtcttg | tccttatttaacatgatgac |
| *RPK1* | *RECEPTOR-LIKE PROTEIN KINASE 1* | AT1G69270 | ggagatgaagaacaccacta | ccgacagctcatcaaaccat |
| *RPK2* | *RECEPTOR-LIKE PROTEIN KINASE 2* | AT3G02130 | gggttttgcgacatttgctg | atggcaaggtcgaagatagg |
| *WOX5* | *WUSCHEL RELATED HOMEOBOX5* | AT3G11260 | gaggcagaaacgtcgtaaaa | aatgtctctatcaccttctc |
